# Clinical confirmation to demonstrate similarity for a biosimilar pegfilgrastim: a 3-way randomized equivalence study for a proposed biosimilar pegfilgrastim versus US-licensed and EU-approved reference products in breast cancer patients receiving myelosuppressive chemotherapy

**DOI:** 10.1186/s40164-018-0114-9

**Published:** 2018-09-06

**Authors:** Kalpna Desai, Priya Misra, Sanyukta Kher, Nirmesh Shah

**Affiliations:** 1 Preclinical and Clinical Programs, Apobiologix, 4100 Weston Road, Toronto, ON M9L 2Y6 Canada; 2 Regulatory Affairs, Apobiologix, 4100 Weston Road, Toronto, ON M9L 2Y6 Canada; 3 Medical Affairs, Apobiologix, 2400 N. Commerce Parkway, Suite 300, Weston, FL 33326 USA

**Keywords:** Biosimilar, Pegfilgrastim, Chemotherapy-induced neutropenia, Severe neutropenia, Febrile neutropenia, Absolute neutrophil count

## Abstract

**Background:**

Chemotherapy-induced neutropenia is a common result of myelosuppressive chemotherapy treatment. Infections such as febrile neutropenia (FN) are sensitive to the duration of neutropenia as well as the depth of absolute neutrophil count (ANC) at nadir. Filgrastim, a granulocyte colony stimulating factor (G-CSF), can stimulate the function of mature neutrophils. Pegfilgrastim, a long-acting form of filgrastim, has been shown to reduce FN to a greater extent compared to filgrastim. G-CSF agents have been recommended for prophylactic administration with chemotherapy. Apotex developed a proposed pegfilgrastim biosimilar. This study was conducted to confirm that no clinically meaningful efficacy or safety differences exist between Apotex’s proposed biosimilar and its reference product.

**Methods:**

589 breast cancer patients were randomized and dosed with the proposed pegfilgrastim biosimilar, US-licensed pegfilgrastim reference product, or EU-approved pegfilgrastim reference product. The primary endpoint assessed was the duration of severe neutropenia (DSN) and secondary endpoints included rate of FN and ANC nadir.

**Results:**

Data showed that the mean DSN, the primary endpoint measured, was comparable across all three treatments. The *As Treated* arm had a 95% confidence interval within the equivalence range for the proposed pegfilgrastim biosimilar with the US-licensed and EU-approved pegfilgrastim reference products. Secondary endpoints, which included depth and peak of ANC nadir, time to ANC recovery post-nadir and rates of FN, also showed similarity between the three different treatment groups. The adverse event incidence was similar across treatment arms and there were no unexpected safety events.

**Conclusions:**

Overall, these results show that the proposed pegfilgrastim biosimilar is similar to Amgen’s US-licensed and EU-approved pegfilgrastim reference products with regard to the clinical efficacy and safety endpoints assessed.

*Trial registration* EMA: European Union Clinical Trials Register: (https://www.clinicaltrialsregister.eu/ctr-search/search?query=eudract_number:2011-002678-21) Eudract # 2011-002678-21 Registered: 01/10/2012

## Background

Myelosuppressive chemotherapy treatment commonly results in chemotherapy-induced neutropenia (CIN), a complication associated with a high risk of morbidity, mortality, and hospitalization [1]. After a course of chemotherapy, the duration of neutropenia and depth of the absolute neutrophil count (ANC) at nadir, correlates with the development of infections including febrile neutropenia (FN), sepsis and their morbidities [2, 3].

Filgrastim, a granulocyte colony stimulating factor (G-CSF), stimulates hematopoietic cells, promoting growth, proliferation, differentiation, and maturation of neutrophil precursors. Filgrastim also enhances the function of mature neutrophils by increasing phagocytic activity and antibody-dependent cell-mediated cytotoxicity [4, 5].

The addition of a polyethylene glycol moiety to filgrastim results in the long-acting form of pegfilgrastim, which requires only once-per-cycle administration for CIN management. Pegfilgrastim retains the same biological activity as filgrastim and binds to the same G-CSF receptor. In both experimental animals and healthy human volunteers, pegfilgrastim showed decreased renal clearance and increased plasma half-life compared with unpegylated filgrastim, allowing pegfilgrastim a sustained pharmacological effect [6]. In clinical trials, patients receiving pegfilgrastim experienced a lower incidence of FN than patients receiving filgrastim [7].

Several international clinical guidelines [European Organization for Research and Treatment of Cancer (EORTC), American Society for Clinical Oncology (ASCO), and National Comprehensive Cancer Network (NCCN)] recommend prophylactic administration of G-CSF (filgrastim or pegfilgrastim) with chemotherapy associated with a high risk (> 20%) of FN and supportive G-CSF therapy with chemotherapy regimens associated with intermediate frequency (10–20%) of FN when additional predisposing factors exist for the patient (e.g., age > 65 years, experience of previous episode(s) of FN, or advanced stage of disease).

Apotex, Inc. and Intas Pharmaceuticals Limited have codeveloped a proposed pegfilgrastim biosimilar product to the US-licensed and EU-approved reference product Neulasta^®^, marketed by Amgen, Inc. State-of-the-art analytical techniques previously demonstrated analytical similarity between the proposed pegfilgrastim biosimilar and the US-licensed pegfilgrastim reference product [8]. In addition, nonclinical primary pharmacodynamic (PD) repeat-dose toxicity/toxicokinetics, local tolerance studies, and a two-way cross-over Phase I pharmacokinetic/pharmacodynamic (PK/PD) study in healthy volunteers showing no unexpected safety events and no clinically relevant PK/PD differences between the proposed pegfilgrastim biosimilar and the US-licensed reference product provides further evidence supporting a conclusion that the proposed pegfilgrastim biosimilar is biosimilar to the reference product [9].

This article presents evidence from a head-to-head clinical confirmatory study comparing efficacy and safety (including immunogenicity) of the proposed pegfilgrastim biosimilar with the reference product (commercially available US-licensed and EU-approved Neulasta^®^) in patients with early breast cancer receiving chemotherapy.

## Methods

### Patient eligibility

Female patients ≥ 18 years of age with Stage IIA, IIB or IIIA breast cancer, who were suitable and intended to undergo adjuvant treatment with TAC (docetaxel, doxorubicin, cyclophosphamide) chemotherapy were eligible for this study. Inclusion criteria included: patients within 60 days of complete surgical resection of the primary breast tumor, either lumpectomy or mastectomy, with sentinel lymph node biopsy or axillary dissection, with clear margins for both invasive and ductal carcinoma in situ (DCIS); Eastern Cooperative Oncology Group (ECOG) performance status ≤ 2; ANC ≥ 1.5 × 10^9^/L; platelet count ≥ 100 × 10^9^/L; adequate renal function (serum creatinine < 1.5 × upper limit of normal [ULN]) and hepatic function (bilirubin < ULN, transaminases and alkaline phosphatase [AP] < 1.5 × ULN); normal cardiac function evidenced by a left ventricle ejection fraction (LVEF) ≥ 55%; no evidence of metastatic disease; and baseline bilateral mammography or other scan to exclude cancer on the contralateral breast. Patients were excluded if they had or received any of the following: (concomitant or prior) except in situ lesion, either ductal or lobular, of the contralateral breast; prior chemotherapy (either adjuvant or neoadjuvant) for this incidence of breast cancer; history of uncontrolled cardiac disease; immunotherapy, hormonal therapy (e.g., tamoxifen or aromatase inhibitors), or Herceptin^®^ (trastuzumab) concurrently or within 30 days of screening; concurrent radiation therapy; investigational therapy concurrently or within 30 days of screening; peripheral neuropathy > Grade 1; major organ allograft or condition requiring chronic immunosuppression; or history of other malignancy within the last 5 years (except cured basal cell carcinoma of skin, carcinoma in situ of uterine cervix, or DCIS).

### Study design and treatment

This was a confirmatory, randomized, controlled, assessor-blinded, multicenter study comparing efficacy and safety of the proposed pegfilgrastim biosimilar to the US-licensed pegfilgrastim reference product and the EU-approved pegfilgrastim reference product conducted in 11 Central and Eastern European countries. The study consisted of three phases: a screening period ≤ 3 weeks; an 18-week active treatment period including 6, 3-week cycles of TAC chemotherapy (docetaxel 75 mg/m^2^, doxorubicin 50 mg/m^2^, and cyclophosphamide 500 mg/m^2^); and a safety follow-up period ≤ 30 weeks following the completion of the TAC regimen. Eligible patients were randomized to either the proposed pegfilgrastim biosimilar (APO-Peg), US-licensed pegfilgrastim reference product (US-Neulasta^®^) or EU-approved pegfilgrastim reference product (EU-Neulasta^®^) in a 2:1:1 ratio. Premedication with dexamethasone and ondansetron was initiated before administration of each chemotherapy cycle. Each patient received 6 doses of pegfilgrastim (6 mg/0.6 mL pre-filled syringe for each) for each chemotherapy cycle, administered as subcutaneous injection on Day 2 of each cycle (at least 24 h after chemotherapy).

In Cycle 1, blood samples were collected for complete blood counts with differentials on Day 0, 1, 3, 5, 6, 7, and every day until ANC recovery post-nadir to ≥ 2.0 × 10^9^/L, or up to Day 15 if recovery did not occur earlier. Blood samples for ANC measurements in Cycles 2–6 were collected mainly to assess safety. Blood samples were collected during the screening, treatment, and follow-up periods for immunogenicity testing.

### Assessments

The primary efficacy endpoint was the duration of severe neutropenia (DSN), defined as ANC below 0.5 × 10^9^/L. Secondary endpoints included: depth and peak of ANC nadir in Cycle 1; time to the ANC recovery post-nadir in Cycle 1; rates of FN (defined as a single temperature ≥ 38.3 °C or temperature ≥ 38.0 °C for over 1 h and ANC less than 0.5 × 10^9^/L or less than 1 × 10^9^/L and predicted decline to ≤ 0.5 × 10^9^/L over the next 48 h) by cycle and across all cycles. Safety assessment included: the incidence of adverse events (AEs) classified by system organ class, preferred term, severity, and relationship to study medication; injection site reactions; vital signs; presence of antibodies to pegfilgrastim; and abnormal clinical laboratory results.

### Statistical analyses

The inference for equivalence was based on the two-sided 95% confidence interval (CI) for the difference in means between the proposed pegfilgrastim biosimilar and the US-licensed or EU-approved pegfilgrastim reference product for DSN in Cycle 1 (in days). Equivalence was defined as a CI range of [− 0.5 days, + 0.5 days]. The two-sided 95% CIs were derived from a one-way ANOVA model accounting for the treatment effect. For statistical analyses, treatment groups were defined as follows:

*Full analysis set (FAS)-As Randomized* included all enrolled subjects who were randomized, received at least one dose of active treatment, and who had any follow-up data for the primary endpoint variables. Treatment assignment for subjects was based on the treatment they were randomized to (*As Randomized* allocation). *FAS-As Randomized* represents the prespecified primary analysis set used for efficacy endpoints.

*FAS-As Treated* included all enrolled subjects who were randomized and received at least one dose of active treatment, and who had any follow-up data for the primary endpoint variables. Treatment assignment for subjects was based on the treatment they received in each cycle instead of the treatment they were randomized to receive.

*Safety analysis set, sensitivity* included all subjects who received at least one dose of active treatment. All subjects who received APO-Peg at any time during the treatment period (regardless of randomized treatment group) were included in the APO-Peg group for this data set. Subjects who did not receive APO-Peg at any time were assigned to their randomized treatment group.

*Per protocol (PP) analysis set* included all enrolled subjects who were randomized and received at least one dose of active treatment, and who had any follow-up data for the primary endpoint variables. Subjects with protocol deviations that impacted the integrity of the primary endpoint data and the safety/well-being of the subject in Cycle 1 were excluded from the PP (Cycle 1) analysis for the primary endpoint. Additionally, subjects with protocol deviations affecting the integrity of the data and the endpoint of the efficacy/safety analysis and well-being of the subject during Cycles 2–6 were excluded from the PP (All Cycles) analyses.

## Results

### Patients

**Fig. 1:**
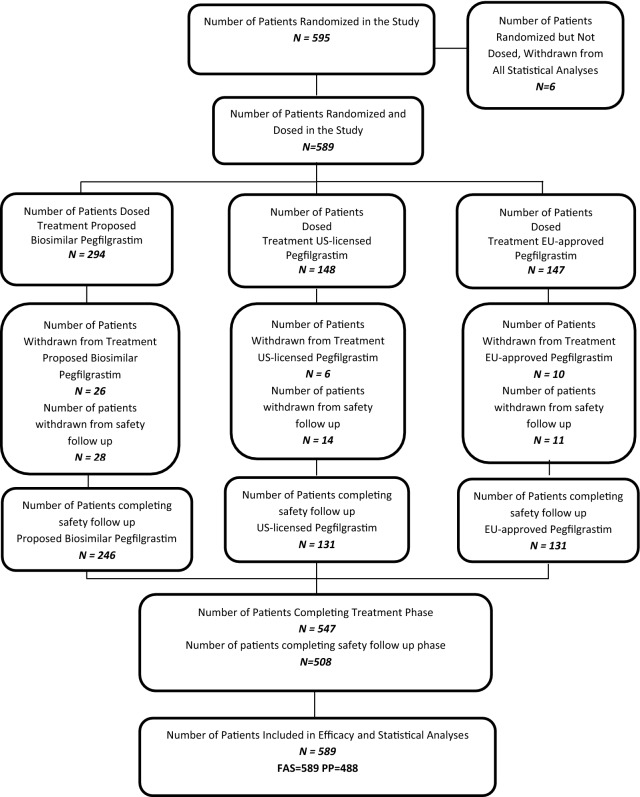
Disposition of patients as randomized: distribution of randomized patients into three arms (the proposed pegfilgrastim biosimilar, US-licensed pegfilgrastim reference product, an EU-approved pegfilgrastim reference product), including the number of patients that withdrew and completed treatment and safety follow up

A total of 595 patients were randomized from 56 investigational centers in 11 countries. All subjects were suitable for neoadjuvant TAC treatment (all chemotherapy naïve subjects: 41.8% Stage IIa; 27.7% Stage 27.7%; and 30.6% Stage IIIa). Patient demographics and breast cancer history were consistent across the treatment arms (Table 1). Of those randomized, 589 patients were dosed, with 547 (92.9%) completing 6 cycles of treatment and 42 (7.1%) discontinuing the study (Fig. 1). Some subjects who withdrew from the treatment phase were followed in the safety follow-up phase.

**Table 1:** Demographic and breast cancer history data—FAS (As Randomized)

	Proposed biosimilar pegfilgrastim (APO-Peg) (*N *= 294)	US-licensed pegfilgrastim reference product (US-Neulasta^®^) (*N *= 148)	EU-approved pegfilgrastim reference product (EU-Neulasta^®^) (*N *= 147)	Total (*N *= 589)
Demographic data				
Female, n (%)	*294* (100)	*148* (100)	*147* (100)	*589* (100)
Age (years)				
Mean (SD)	51.9 (10.0)	51.4 (10.4)	51.5 (10.2)	51.7 (10.1)
Median (min, max)	52.0 (24.0, 75.0)	52.0 (27.0, 80.0)	53.0 (22.0, 77.0)	52.0 (22.0, 80.0)
Race, n (%) Caucasian	*294* (100.0)	*148* (100.0)	*147* (100.0)	*589* (100.0)
Body weight (kg)				
Mean (SD)	73.88 (14.4)	72.01 (14.1)	72.61 (12.9)	73.09 (14.0)
Median (min, max)	73.0 (40.0, 120.0)	70.0 (40.0, 118.0)	70.0 (48.0, 119.0)	72.0 (40.0, 120.0)
Body height (cm)				
Mean (SD)	162.5 (6.8)	162.7 (6.6)	162.6 (6.4)	162.6 (6.6)
Median (min, max)	163.0 (140.0, 180.0)	163.0 (142.0, 180.0)	163.0 (148.0, 183.0)	163.0 (140.0, 183.0)
Breast cancer history				
Tumor parameter				
Staging IIA	129 (43.9)	59 (39.9)	58 (39.5)	246 (41.8)
Staging IIB	79 (26.9)	40 (27.0)	44 (29.9)	163 (27.7)
Staging IIIA	86 (29.3)	49 (33.1)	45 (30.6)	180 (30.6)

T1, T2, T3 means the size and/or extent of the primary tumor stage (increasing order from 1 to 3)

### Comparative efficacy

As a sensitive measure of efficacy [10], the assessment of DSN in Cycle 1 (versus subsequent cycles) was chosen as the primary endpoint. Overall, the mean DSN in Cycle 1 is comparable across treatments and essentially the same between the FAS *As Treated* and *As Randomized* populations (Table 2). For the *As Randomized* population, the 95% CI of the difference in mean DSN in Cycle 1 between the proposed pegfilgrastim biosimilar and US-licensed pegfilgrastim reference product was slightly outside the equivalence margin, but for the proposed pegfilgrastim biosimilar and EU-approved pegfilgrastim reference product was contained within the pre-defined margin (Table 2). Given that sampling of ANC for determination of DSN in a clinical setting is conducted on a daily basis, as in this study, the breach of 0.01 days, which equates to approximately 14.4 min from the upper limit of the equivalence range (0.51 days), is not considered clinically significant. The results for the *As Treated* population showed a 95% CI within the equivalence range for the proposed pegfilgrastim biosimilar between both the US-licensed and EU-approved pegfilgrastim reference products, confirming similar efficacy (Table 2).

**Table 2:** Summary of DSN in cycle 1 (FAS-As Randomized and FAS-As Treated)

Analysis Set Statistic	Proposed biosimilar pegfilgrastim (APO-Peg)	US-licensed pegfilgrastim reference product (US-Neulasta^®^)	EU-approved pegfilgrastim reference product (EU-Neulasta^®^)	(APO-Peg)-minus (US-Neulasta^®^)	(APO-Peg)-minus (EU-Neulasta^®^)	(EU-Neulasta^®^) minus (US-Neulasta^®^)
FAS (As Randomized)						
N	294	148	147	N/A	N/A	N/A
LS Mean in days	1.6	1.4	1.6	0.2	0.02	0.2
Median (Min, Max) in days	2.0 (0, 10)	1.0 (0, 5)	2.0 (0, 10)	N/A	N/A	N/A
95% CI in days	1.47 to 1.79	1.17 to 1.61	1.38 to 1.83	− 0.03 to 0.51	− 0.25 to 0.30	− 0.10 to 0.53
FAS (As Treated)						
N	*298*	*147*	*144*	N/A	N/A	N/A
LS Mean in days	1.6	1.4	1.6	0.2	-0.01	0.2
Median (Min, Max) in days	1.5 (0, 10)	1.0 (0, 5)	2.0 (0, 10)	N/A	N/A	N/A
95% CI in days	1.46 to 1.77	1.17 to 1.61	1.41 to 1.86	− 0.04 to 0.50	− 0.29 to 0.26	− 0.07 to 0.56

Similarity between the proposed pegfilgrastim biosimilar and the EU-approved and US-licensed reference products was also supported by comparative results between all treatment arms for all secondary efficacy endpoints (Table 3 and data not shown). The rate of FN (*As Randomized*) was similar between treatment arms with the highest incidence occurring in Cycle 1 (as expected) and decreasing in subsequent treatment cycles (Table 3). In addition, the mean ANC values showed similar results among the three treatment arms [overall mean peak ANC of 28.9 × 10^9^/L (day 3.1–3.4); overall mean ANC nadir of 0.5 × 10^9^/L (day 7.1–7.3), and recovery of ANC (≥ 2.0 × 10^9^/L; day 9.2–9.5)] (Table 4).

**Table 3:** Rates of FN by cycle following treatment (FAS—As Randomized)

Cycle	Proposed biosimilar pegfilgrastim (APO-Peg)	US-licensed pegfilgrastim reference product (US-Neulasta^®^)	EU-approved pegfilgrastim reference product (EU-Neulasta^®^)	Total
Cycle 1				
n/N (%)	15/294 (5.1)	6/148 (4.1)	5/147 (3.4)	26/589 (4.4)
95% CI	2.9–8.3	1.5–8.6	1.1–7.8	2.9–6.4
Cycle 2				
n/N (%)	0/288 (0.0)	1/146 (0.7)	0/144 (0.0)	1/578 (0.2)
95% CI	0.0–1.3	0.0–3.8	0.0–2.5	0.0–1.0
Cycle 3				
n/N (%)	0/285 (0.0)	0/146 (0.0)	1/144 (0.7)	1/575 (0.2)
95% CI	0.0–1.3	0.0–2.5	0.0–3.8	0.0–1.0
Cycle 4				
n/N (%)	0/284 (0.0)	0/144 (0.0)	0/144 (0.0)	0/572 (0.0)
95% CI	0.0–1.3	0.0–2.5	0.0–2.5	0.0–0.6
Cycle 5				
n/N (%)	2/276 (0.7)	0/143 (0.0)	0/142 (0.0)	2/561 (0.4)
95% CI	0.1–2.6	0.0–2.5	0.0–2.6	0.0–1.3
Cycle 6				
n/N (%)	0/270 (0.0)	0/143 (0.0)	0/139 (0.0)	0/552 (0.0)
95% CI	0.0–1.4	0.0–2.5	0.0–2.6	0.0–0.7
Overall				
n/N (%)	17/294 (5.8)	7/148 (4.7)	5/147 (3.4)	29/589 (4.9)
95% CI	3.4–9.1	1.9–9.5	1.1–7.8	3.3–7.0

**Table 4:** Mean ANC values for planned sampling days in cycle 1 (FAS-As Randomized and FAS-As Treated)

Characteristics	Proposed biosimilar pegfilgrastim (APO-Peg) *N *= 294	US-licensed pegfilgrastim reference product (US-Neulasta^®^) *N *= 148	EU-approved pegfilgrastim reference product (EU-Neulasta^®^) *N *= 147	Total *N *= 589
FAS-As Randomized				
Day on which the peak ANC value was reached				
N	294	148	147	589
Mean (SD)	3.4 (2.3)	3.1 (0.6)	3.1 (1.4)	3.2 (1.8)
Median	3.0	3.0	3.0	3.0
Minimum–maximum	1–24	3–9	1–18	1–24
95% CIs of mean	3.1–3.6	3.0–3.2	2.9–3.4	3.1–3.4
Peak ANC value (× 10^9^/L)				
N	294	148	147	589
Mean (SD)	28.4 (9.5)	29.9 (10.2)	28.7 (9.3)	28.9 (9.6)
Median	27.7	29.4	27.9	28.0
Minimum–maximum	3.9–60.5	11.3–88.0	5.8–55.9	3.9–88.0
95% CIs of mean	27.3–29.5	28.3–31.6	27.2–30.2	28.1–29.7
Day on which the depth of ANC nadir was reached				
N	294	148	147	589
Mean (SD)	7.1 (1.8)	7.1 (0.6)	7.3 (2.1)	7.1 (1.7)
Median	7.0	7.0	7.0	7.0
Minimum–maximum	1–21	6–11	6–21	1–21
95% CIs of mean	6.9–7.3	7.0–7.2	7.0–7.7	7.0–7.3
Depth of ANC nadir (× 10^9^/L)				
N	294	148	147	589
Mean (SD)	0.6 (1.1)	0.4 (0.6)	0.4 (0.7)	0.5 (0.9)
Median	0.2	0.2	0.1	0.2
Minimum–maximum	0.0–9.8	0.0–3.4	0.0–6.0	0.0–9.8
95% CIs of mean	0.4–0.7	0.3–0.5	0.3–0.5	0.4–0.6
Day on which recovery of ANC was reached				
N	267	136	137	540
Mean (SD)	9.4 (2.0)	9.5 (2.1)	9.2 (1.0)	9.4 (1.8)
Median	9.0	9.0	9.0	9.0
Minimum–maximum	6–25	8–22	8–13	6–25
95% CIs of mean	9.2–9.7	9.1–9.8	9.0–9.4	9.2–9.5

Characteristics	Proposed biosimilar pegfilgrastim (APO-Peg) *N *= 298	US-licensed pegfilgrastim reference product (US-Neulasta^®^) *N *= 147	EU-approved pegfilgrastim reference product (EU-Neulasta^®^) *N *= 147	Total *N *= 589
FAS-As Treated				
Day on which the peak ANC value was reached				
N	298	147	144	589
Mean (SD)	3.4 (2.3)	3.1 (0.5)	3.1 (1.4)	3.2 (1.8)
Median	3.0	3.0	3.0	3.0
Minimum–maximum	1–24	3–9	1–18	1–24
95% CIs of mean	3.1–3.6	3.0–3.2	2.9–3.4	3.1–3.4
Peak ANC value (× 10^9^/L)				
N	298	147	144	589
Mean (SD)	28.5 (9.6)	29.7 (10.2)	28.8 (9.2)	28.9 (9.6)
Median	27.7	28.6	28.3	28.0
Minimum–maximum	3.9–60.5	11.3–88.0	5.8–55.9	3.9–88.0
95% CIs of mean	27.4–29.6	28.1–31.4	27.3–30.4	28.1–29.7
Day on which the depth of ANC nadir was reached				
N	298	147	144	589
Mean (SD)	7.1 (2.0)	7.1 (0.6)	7.2 (1.8)	7.1 (1.7)
Median	7.0	7.0	7.0	7.0
Minimum–maximum	1–21	6–11	6–21	1–21
95% CIs of mean	6.9–7.4	7.0–7.2	6.9–7.5	7.0–7.3
Depth of ANC nadir (× 10^9^/L)				
N	298	147	144	589
Mean (SD)	0.6 (1.1)	0.4 (0.6)	0.4 (0.7)	0.5 (0.9)
Median	0.2	0.2	0.1	0.2
Minimum–maximum	0.0–9.8	0.0–3.4	0.0–6.0	0.0–9.8
95% CIs of mean	0.4–0.7	0.3–0.5	0.3–0.5	0.4–0.6
Day on which recovery of ANC was reached				
N	270	135	135	540
Mean (SD)	9.4 (2)	9.4 (2.1)	9.2 (1.0)	9.4 (1.8)
Median	9.0	9.0	9.0	9.0
Minimum–maximum	6–25	8–22	8–13	6–25
95% CIs of mean	9.2–9.7	9.1–9.8	9.0–9.4	9.2–9.5

### Comparative safety

The number of AEs in the three treatment arms were similar, and the majority were considered to be mild to moderate in severity (Table 5). For this analysis, any subject receiving the proposed biosimilar in any cycle (regardless of randomization) was included in the APO-Peg arm. Out of 589 subjects across the treatment arms, *539* (91.5%) reported at least one AE in the treatment phase (89.7% in the APO-Peg arm; 94.8% in the US-Neulasta arm; 92.8% in the EU-Neulasta arm; Table 5).

**Table 5:** Frequency of most common adverse events (≥ 5%) in treatment period—safety analysis set sensitivity

System organ class	Preferred term	Proposed biosimilar pegfilgrastim (APO-Peg) *N *= 329	US-licensed pegfilgrastim reference product (US-Neulasta^®^) N = 135	EU-approved pegfilgrastim reference product (EU-Neulasta^®^) N = 125	Total N = 589
Any AE		295 (89.7)	128 (94.8)	116 (92.8)	539 (91.5)
Blood and lymphatic system disorders	All PTs	185 (56.2)	88 (65.2)	73 (58.4)	346 (58.7)
	Anaemia	14 (4.3)	8 (5.9)	8 (6.4)	30 (5.1)
	Febrile Neutropenia	15 (4.6)	7 (5.2)	4 (3.2)	26 (4.4)
	Leukocytosis	22 (6.7)	17 (12.6)	13 (10.4)	52 (8.8)
	Leukopenia	63 (19.1)	40 (29.6)	41 (32.8)	144 (24.4)
	Neutropenia	169 (51.4)	79 (58.5)	63 (50.4)	311 (52.8)
	Neutrophilia	14 (4.3)	13 (9.6)	11 (8.8)	38 (6.5)
	Thrombocytopenia	12 (3.6)	5 (3.7)	16 (12.8)	33 (5.6)
Ear and labyrinth disorders	All PTs	17 (5.2)	11 (8.1)	15 (12.0)	43 (7.3)
	Vertigo	17 (5.2)	9 (6.7)	14 (11.2)	40 (6.8)
Gastrointestinal disorders	All PTs	176 (53.5)	81 (60.0)	79 (63.2)	336 (57.0)
	Abdominal pain	19 (5.8)	8 (5.9)	11 (8.8)	38 (6.5)
	Abdominal pain Upper	20 (6.1)	12 (8.9)	17 (13.6)	49 (8.3)
	Constipation	8 (2.4)	5 (3.7)	7 (5.6)	20 (3.4)
	Diarrhoea	54 (16.4)	32 (23.7)	34 (27.2)	120 (20.4)
	Dyspepsia	9 (2.7)	8 (5.9)	11 (8.8)	28 (4.8)
	Nausea	145 (44.1)	67 (49.6)	65 (52.0)	277 (47.0)
	Stomatitis	20 (6.1)	10 (7.4)	5 (4.0)	35 (5.9)
	Vomiting	44 (13.4)	18 (13.3)	27 (21.6)	89 (15.1)
General disorders and administration site conditions	All PTs	147 (44.7)	71 (52.6)	69 (55.2)	287 (48.7)
	Asthenia	81 (24.6)	41 (30.4)	31 (24.8)	153 (26.0)
	Fatigue	43 (13.1)	18 (13.3)	32 (25.6)	93 (15.8)
	Malaise	9 (2.7)	8 (5.9)	5 (4.0)	22 (3.7)
	Oedema Peripheral	15 (4.6)	10 (7.4)	9 (7.2)	34 (5.8)
	Pyrexia	22 (6.7)	12 (8.9)	18 (14.4)	52 (8.8)
Metabolism and nutrition disorders	All PTs	28 (8.5)	16 (11.9)	26 (20.8)	70 (11.9)
	Decreased Appetite	12 (3.6)	9 (6.7)	17 (13.6)	38 (6.5)
Musculoskeletal and connective tissue disorders	All PTs	166 (50.5)	77 (57.0)	80 (64.0)	323 (54.8)
	Arthralgia	13 (4.0)	8 (5.9)	10 (8.0)	31 (5.3)
	Bone pain	149 (45.3)	71 (52.6)	70 (56.0)	290 (49.2)
	Myalgia	28 (8.5)	19 (14.1)	15 (12.0)	62 (10.5)
Nervous system disorders	All PTs	119 (36.2)	63 (46.7)	53 (42.4)	235 (39.9)
	Dizziness	68 (20.7)	24 (17.8)	21 (16.8)	113 (19.2)
	Headache	67 (20.4)	38 (28.1)	31 (24.8)	136 (23.1)
	Hypoesthesia	9 (2.7)	9 (6.7)	6 (4.8)	24 (4.1)
Respiratory, thoracic and mediastinal disorders	All PTs	31 (9.4)	16 (11.9)	19 (15.2)	66 (11.2)
	Oropharyngeal Pain	14 (4.3)	8 (5.9)	6 (4.8)	28 (4.8)
Skin and subcutaneous tissue disorders	All PTs	92 (28.0)	45 (33.3)	46 (36.8)	183 (31.1)
	Alopecia	78 (23.7)	36 (26.7)	37 (29.6)	151 (25.6)
	Scar pain	3 (0.9)	0 (0.0)	7 (5.6)	10 (1.7)

Two of the most common AEs were neutropenia in (52.8% of subjects overall; 51.4% in the APO-Peg arm; 58.5% in the US-Neulasta arm; 50.4% in the EU-Neulasta arm) and nausea (47.0% of subjects overall; 44.1% in the APO-Peg arm; 49.6% in the US-Neulasta arm; 52.0% in the EU-Neulasta arm) (Table 5). In addition, bone pain, a common event associated with pegfilgrastim [12] thought to be directly related to treatment with filgrastim, occurred at a similar frequency in the three treatment groups (45.3% in the APO-Peg arm; 52.6% in the US-Neulasta arm; 56.0% in the EU-Neulasta arm; Table 5) and none of these events were reported as a serious adverse event (SAE), or led to subject withdrawal.

One subject randomized to US-licensed pegfilgrastim reference product but receiving the proposed pegfilgrastim biosimilar died due to disease progression. Five additional life-threatening events were reported in the APO-Peg treatment arm (3 FN, 1 pancytopenia, and 1 pulmonary embolism). None of these events were related to the study drug. The incidence of immunogenicity was low and similar across treatment arms, including no apparent clinically meaningful effects associated with anti-drug antibodies (ADAs) or neutralizing antibodies (data not shown).

## Discussion

The term *biosimilarity*, defined in section 351(i) of the Public Health Services (PHS) Act [11], means that the biological product is “highly similar to the reference product notwithstanding minor differences in clinically inactive components; and there are no clinically meaningful differences between the biological product and the reference product in terms of the safety, purity, and potency of the product” [12]. A demonstration that a product is biosimilar to its reference product is established analytically and then confirmed clinically in patients to show that the efficacy and safety profiles of the proposed biosimilar and reference product are comparable. This clinical confirmatory study is not meant to demonstrate the safety and efficacy of the biosimilar a priori, but to provide additional evidence that the proposed biosimilar and the reference product are similar giving the same clinical results as the reference biologic in a head-to-head study.

Building on prior work demonstrating that the Apotex/Intas proposed biosimilar pegfilgrastim is analytically highly similar to US-licensed pegfilgrastim [8], as well similar with regards to PK/PD and safety in healthy volunteers [9], results from this clinical confirmatory study in breast cancer patients further support the biosimilarity of the proposed biosimilar pegfilgrastim to the US-licensed and EU-licensed pegfilgrastim reference product.

The proposed biosimilar showed comparable mean DSN in Cycle 1 to both the US-licensed and EU-approved pegfilgrastim reference products, and the data are consistent with the published data for these reference products. While the 95% CI of the difference in mean DSN in Cycle 1 for the *As Randomized* population was slightly outside the pre-defined equivalence margin of ± 0.5 days, prior data suggest that this range is very conservative. An equivalence range of ± 1 day, consistent with the usual daily sampling of ANC, has commonly been used in other similar studies [13, 14]. Given that sampling of ANC for determination of DSN in a clinical setting is conducted on a daily basis, as in this study, the breach of 0.01 days, which equates to approximately 14.4 min from the upper limit of the equivalence range (0.51 days), is not considered clinically significant. In outpatient practice, neutrophils are measured on the day of chemotherapy administration, without considerations about when the nadir occurred, or recovery of ANC. Even in a hospitalized setting, with patients at risk of developing febrile neutropenia, and monitored daily, a difference of 14.4 min in DSN would not have any clinical impact.

The mean DSN in the absence of G-CSF in breast cancer patients treated with doxorubicin and docetaxel is expected to be 6–7 days [15], while prophylactic treatment with a single fixed 6-mg dose of pegfilgrastim is expected to reduce the mean DSN in Cycle 1 to approximately 1.8 days [14]. Thus, the treatment effect of pegfilgrastim in the reduction of the mean DSN in Cycle 1 can be expected to be between 4.2 and 5.2 days, and as such, an equivalence interval of ± 0.5 days should ensure that at least 88%–90% of the effect size (i.e., pegfilgrastim reduction in DSN) is retained, a more stringent requirement than typically applied in non-inferiority or equivalence studies.

Similarity between the proposed pegfilgrastim biosimilar and the US-licensed and EU-approved pegfilgrastim reference products was also supported by the results from all secondary efficacy analyses including the depth and peak of ANC nadir in Cycle 1, the time to ANC recovery post-nadir in Cycle 1, the rates of FN by cycle and across cycles, and the ANC time profile in Cycle 1.

The AE incidence was similar across treatment arms and there were no unexpected safety events for the proposed pegfilgrastim biosimilar. The incidence of immunogenicity was low and similar across treatment arms, including no apparent clinically meaningful effects associated with anti-drug antibodies (ADAs) or neutralizing antibodies. Overall, these results contribute to the totality of the evidence collected to support a conclusion of biosimilarity between the proposed biosimilar pegfilgrastim and the reference product. Just like originator biologics, post marketing surveillance data, from the countries where the product is approved, is being continually monitored and the results are no different from those found with other pegfilgrastim products.

## Conclusions

This head-to-head, randomized, clinical confirmatory study of a proposed pegfilgrastim biosimilar demonstrates that the proposed pegfilgrastim is similar to Amgen’s US-licensed and EU-approved pegfilgrastim reference products, with regard to safety and efficacy, in the treatment of chemotherapy-induced neutropenia in breast cancer patients receiving myelosuppressive chemotherapy.

## Abbreviations

ADA: anti-drug antibodies
AE: adverse event
ANC: absolute neutrophil count
AP: alkaline phosphatase
APO: Apotex
ASCO: American Society for Clinical Oncology
CI: confidence interval
CIN: chemotherapy-induced neutropenia
DCIS: ductal carcinoma in situ
DSN: duration of severe neutropenia
ECOG: Eastern Cooperative Oncology Group
EORTC: European Organization for Research and Treatment of Cancer
EU: European Union
FAS: Full analysis set
FN: febrile neutropenia
G-CSF: granulocyte colony stimulating factor
LS: least square
LVEF: left ventricle ejection fraction
NCCN: National Comprehensive Cancer Network
PD: pharmacodynamic
PHS: Public Health Services
PK: pharmacokinetic
PP: per protocol
PT: proposed term
TAC: docetaxel, doxorubicin, cyclophosphamide
SAE: serious adverse event
SD: standard deviation
ULN: upper limit of normal
US: United States
